# Genome‐Wide CRISPR/Cas9 Library Screening Revealed Dietary Restriction of Glutamine in Combination with Inhibition of Pyruvate Metabolism as Effective Liver Cancer Treatment

**DOI:** 10.1002/advs.202202104

**Published:** 2022-10-30

**Authors:** Chunxue Yang, Derek Lee, Misty Shuo Zhang, Aki Pui‐Wah Tse, Lai Wei, Macus Hao‐Ran Bao, Bowie Po‐Yee Wong, Cerise Yuen‐Ki Chan, Vincent Wai‐Hin Yuen, Yiling Chen, Carmen Chak‐Lui Wong

**Affiliations:** ^1^ Department of Pathology The University of Hong Kong Hong Kong P. R. China; ^2^ State Key Laboratory of Liver Research The University of Hong Kong Hong Kong P. R. China; ^3^ School of Public Health (Shenzhen) Sun Yat‐sen University Guangzhou 510275 P. R. China; ^4^ Centre for Oncology and Immunology Hong Kong Science Park Hong Kong P. R. China; ^5^ Guangdong‐Hong Kong Joint Laboratory for RNA Medicine Sun Yat‐sen University Guangzhou 510120 P. R. China

**Keywords:** CRISPR/Cas9 library screening, dietary intervention, glutamine depletion, hepatocellular carcinoma, pyruvate metabolism

## Abstract

Hepatocellular carcinoma (HCC) is the second most lethal cancer worldwide. Glutamine is an essential, extracellular nutrient which supports HCC growth. Dietary glutamine deficiency may be a potential therapeutic approach for HCC. HCC cells overcome metabolic challenges by rewiring their metabolic pathways for rapid adaptations. The efficiency of dietary glutamine deficiency as HCC treatment is examined and the adaptation machinery under glutamine depletion in HCC cells is unraveled. Using genome‐wide CRISPR/Cas9 knockout library screening, this study identifies that pyruvate dehydrogenase *α* (PDHA), pyruvate dehydrogenase *β* (PDHB), and pyruvate carboxylase (PC) in pyruvate metabolism are crucial to the adaptation of glutamine depletion in HCC cells. Knockout of either PDHA, PDHB or PC induced metabolic reprogramming of the tricarboxylic acid (TCA) cycle, disrupts mitochondrial function, leading to the suppression of HCC cell proliferation under glutamine depletion. Surprisingly, dietary glutamine restriction improves therapeutic responses of HCC to PDH or PC inhibitor in mouse HCC models. Stable isotope carbon tracing confirms that PDH or PC inhibitors further disrupt the metabolic rewiring of the TCA cycle induced by dietary glutamine depletion in HCC. In summary, the results demonstrate that pyruvate metabolism acts as novel targetable metabolic vulnerabilities for HCC treatment in combination with a glutamine‐deficient diet.

## Graphical abstract

Herein, the authors identify and functionally characterize pyruvate metabolism as metabolic vulnerabilities for HCC treatment using genome wide CRISPR library screening. Suppressing either pyruvate dehydrogenase (PDH) or pyruvate carboxylase (PC) in pyruvate metabolism leading to metabolic disruption of TCA cycle and sensitizing tumors to the treatment of glutamine dietary restriction.

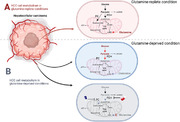

## 1 Introduction

Hepatocellular carcinoma (HCC) originates from hepatocytes, accounts for 90% of all primary liver cancer and is the second leading cause of cancer‐related deaths globally.^[^ ^1^ ^]^ The high therapeutic resistance of HCC increases the difficulty of treatment and leads to the high mortality of HCC patients. The FDA‐approved first‐line drugs for HCC patients, sorafenib and lenvatinib, extends HCC patient survival for only several months.^[^ ^2^ ^]^ Recently, clinical trial results indicated combination treatment of atezolizumab (anti‐PD‐L1) and bevacizumab (anti‐VEGF) achieved improved overall and progression‐free survival benefits compared to sorafenib treatment.^[^ ^3^ ^]^ Cancer cells have high adaptability to depletion of different nutrients by flexibly rewiring their metabolic programs. Understanding the underlying molecular mechanisms enables the identification of new therapeutic strategies targeting the metabolic flexibility and growth of HCC.

Glucose and glutamine are the two most abundant nutrients consumed by cancer cells to support rapid proliferation and tumor growth. As over 90% glucose is converted into lactic acid through aerobic glycolysis, glutamine becomes the main source of carbon and nitrogen for anabolic processes to support cancer growth.^[^ ^4^ ^]^ Glutamine is catabolized into glutamate by glutaminase (GLS) and glutamate is further converted into *α*‐ketoglutarate (*α*‐KG) by glutamate dehydrogenase (GLUD). *α*‐KG fuels the tricarboxylic acid (TCA) cycle for the biosynthesis of carbonous and nitrogenous compounds, for instance nucleotides and non‐essential amino acids to support the rapid proliferation of cancer cells.^[^ ^5^ ^]^

As *α*‐KG is the key intermediate in the TCA cycle, it coordinates the critical metabolic and cellular pathways such as energy metabolism and synthesis of building blocks. Oxidative metabolism of *α*‐KG in the TCA cycle generates adenosine triphosphate (ATP) and produces oxaloacetate, then aspartate for nucleotide and amino acid synthesis.^[^ ^6^ ^]^ The reductive carboxylation of *α*‐KG generates citrate which is converted to acetyl‐CoA for lipid synthesis. Notably, *α*‐KG acts as the co‐substrate for DNA and histone demethylases and therefore determines the epigenetic landscape in cancer cells.^[^ ^7^ ^]^ Due to the multiple roles of *α*‐KG, its replenishment through glutamine is critical to tumor growth. Moreover, glutamine metabolism also maintains the cellular reactive oxygen species (ROS) homeostasis in cancer cells by increasing glutamine oxidation and the production of glutathione (GSH).^[^ ^5^ ^]^ Even though glutamine uptake is increased in cancer cells, the poor vascularization in solid tumors often leads to severe glutamine shortage within the tumor microenvironment.^[^ ^8^ ^]^ Lower glutamine levels were detected in the core regions of melanoma xenografts and transgenic mouse tumors than in the peripheral regions.^[^ ^7^ ^]^

Dietary intervention as a component of cancer treatment is a hotly‐pursued research area and has recently been integrated with various cancer therapies to control tumor progression.^[^ ^9^, ^10^ ^]^ Dietary interventions on normal cells are reversible with minimal toxicity without eliciting permanent damages. Clinical studies implied the potential benefits of ketogenic diet for patients undergoing treatment.^[^ ^11^ ^]^ Methionine restriction diet improves the therapeutic response of colorectal cancer to chemotherapy and radiation through the inhibition of one‐carbon metabolism and nucleotide synthesis.^[^ ^12^ ^]^ However, the understanding of how nutrient composition alters metabolic pathways in tumors and whether diets influence therapeutic outcomes are largely unknown.

“Glutamine addiction” of cancer cells could be best demonstrated from extensive cancer cell death upon withdrawal of glutamine from the culture medium.^[^ ^13^ ^]^ Glutaminase inhibition could significantly suppress breast tumor growth, while lung tumors were resistant to glutaminase inhibition or genetic depletion, suggesting the distinct metabolic output of cancer cells could be affected by different parameters such as the microenvironment.^[^ ^14^, ^15^, ^16^ ^]^ Therefore, we reasoned that glutamine restriction in diets could induce metabolic alterations and enhance the therapeutic response to pharmaceutical treatment for HCC. The CRISPR/Cas9 library screenings have been widely applied for the identification of genes and targets related to cell survival, proliferation, and drug resistance in various models.^[^ ^17^, ^18^ ^]^ Recently, our group has employed genome‐wide CRISPR/Cas9 knockout (KO) library screening to identify the molecular mechanisms of sorafenib resistance and hypoxia adaptation in HCC. We found that serine synthesis pathway (SSP) and cardiolipin synthesis are metabolic vulnerabilities of sorafenib‐resistant and hypoxic HCC cells, respectively.^[^ ^19^, ^20^ ^]^ Therapeutically, targeting the first committed enzyme of the SSP, phosphoglycerate dehydrogenase (PHGDH), sensitized HCC cells to sorafenib treatment^[^ ^19^ ^]^ while targeting protein‐tyrosine phosphatase mitochondrial 1 (PTPMT1), the rate‐limiting enzyme in the cardiolipin synthesis pathway sensitized hypoxic HCC cells to cell death. Currently, we performed genome‐wide CRISPR‐library screening and identified that pyruvate dehydrogenase *α* (PDHA), pyruvate dehydrogenase *β* (PDHB), and pyruvate carboxylase (PC) in pyruvate metabolism are crucial to the adaptation to glutamine depletion. HCC cells shifted reliance on pyruvate metabolism to fuel TCA cycle and maintain mitochondrial function for survival and proliferation. The inhibition of either PDH or PC could sensitize HCC cells to glutamine depletion. Treatment of PDH or PC inhibitor acts synergistically with glutamine depletion or GLS inhibition in suppressing HCC. Importantly, we found that glutamine restriction dietary intervention could improve the therapeutic response of HCC to PDH or PC inhibitor, suggesting that dietary intervention combining with pyruvate metabolism inhibition would be a promising novel therapeutic strategy for HCC treatment.

## 2 Results

### 2.1 CRISPR Library Screening Identified Pyruvate Metabolism to Be Essential for Survival of HCC Cells Under Glutamine Deficiency

**Figure 1:**
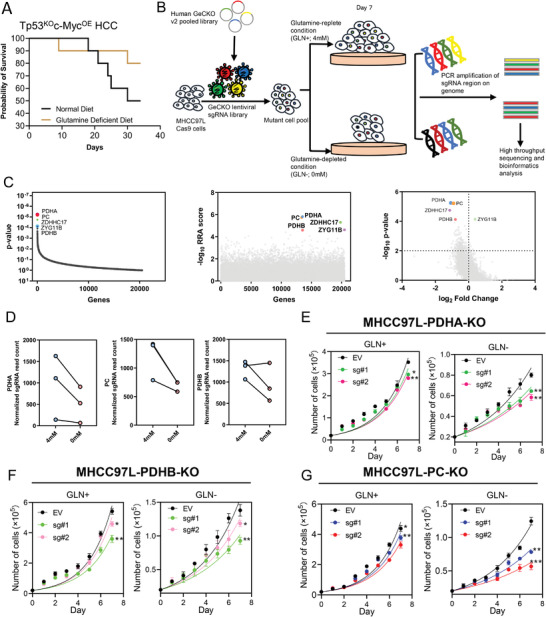
CRISPR/Cas9 library screening identified PDH and PC as critical drivers for glutamine depletion. A) Survival curve of Tp53^KO^c‐Myc^OE^ HCC mice induced by hydrodynamic tail vein injection (HDTVi) of genome editing plasmids and fed with different diets (*n* = 10). B) Schematic diagram illustrates the workflow of genome‐wide CRISPR/Cas9 knockout library screening. C) Pyruvate dehydrogenase *α* (PDHA), pyruvate carboxylase (PC), and pyruvate dehydrogenase *β* (PDHB) were identified as the most significant genes in the library screen. D) The sgRNAs targeting PDHA, PC, and PDHB were negatively selected during glutamine depletion. E–G) Cell proliferation assays demonstrated that knockout (KO) of E) PDHA, F) PDHB, and G) PC significantly suppressed HCC proliferation under glutamine depletion (GLN−). *p < 0.05, **p < 0.01, ***p < 0.001, ****p < 0.0001 versus EV as indicated. Student's *t*‐test. Error bars indicate mean ± SEM (*n* = 3).

To examine whether glutamine deficient diet could be used as a potential therapeutic strategy for HCC treatment, we employed a highly aggressive mouse HCC model involving hydrodynamic tail vein injection (HDTVi) of genome editing plasmids to knock out tumor suppressor Tp53 (Tp53^KO^) and over‐express oncogene c‐Myc (c‐Myc^OE^) in the liver using CRISPR‐Cas9 system and Sleeping Beauty (SB) transposon system, as we previously described.^[^ ^21^ ^]^ HDTVi causes temporary cardiac arrest and a pressurized backflush of plasmids into the liver leading to transfection of hepatocytes which are ultimately transformed into HCC with Tp53^KO^c‐Myc^OE^ genotype. A glutamine deficient diet significantly improved the survival of mice with HCC (**Figure** **1**A). A genome‐wide CRISPR/Cas9 KO screening was performed in human HCC cells MHCC97L cultured in medium with or without glutamine to systematically evaluate the underpinning adaptive mechanisms of glutamine depletion in HCC cells (Figure 1B). A human GeCKO v2 CRISPR library A which contains 65386 unique sgRNAs that targets 19052 protein‐coding genes and 1864 microRNAs were stably transfected into MHCC97L‐Cas9 cells. The mutant cell pool was then cultured in glutamine‐replete (GLN+, 4 mm) or glutamine depleted (GLN−, 0 mm) medium for 7 days (Figure 1B). We hypothesized that KO of genes responsible for adaptation to glutamine depletion will inhibit HCC proliferation in glutamine depleted condition and hence the sgRNAs will be negatively selected. The copy numbers of sgRNAs were evaluated by Next‐Generation Sequencing. After glutamine depletion, we reached ≈400× coverage of library with retention of 95% sgRNAs, ascertaining sufficient read‐depth and library coverage for the screening. GO enrichment analysis showed that pyruvate metabolism was the most important pathway for adaptation to glutamine depletion (Figure S1, Supporting Information). With MAGeCK algorithm, we found that the key enzymes in pyruvate metabolism including PDHA, PC and PDHB ranked as the 1st, 2nd and 5th most important genes for HCC cell survival upon glutamine withdrawal (Figure 1C). PDHA and PDHB are two subunits of pyruvate dehydrogenase (PDH) which converts pyruvate to form acetyl‐CoA. PC converts pyruvate into oxaloacetate which reacts with acetyl‐CoA to synthesize citrate in the TCA cycle.^[^ ^22^ ^]^ All sgRNAs targeting PDH or PC were dramatically decreased in cells cultured in glutamine depleted condition, suggesting that the loss of PDH or PC could sensitize HCC cells to glutamine depletion (Figure 1D).

Next, to further confirm the results from CRISPR/Cas9 library screening, we established PDHA, PDHB and PC stable KO subclones in MHCC97L cells (Figure S2, Supporting Information). The established subclones were cultured in glutamine supplemented (GLN+, 4 mm) or glutamine depleted medium (GLN−, 0 mm). Generally, KO of PDHA, PDHB, and PC significantly suppressed HCC cell proliferation under glutamine deficient condition and mildly suppressed HCC cell proliferation with glutamine (Figure 1E–G). Together, these data suggested that cancer cells are dependent on pyruvate metabolism for survival and proliferation when glutamine is withdrawn.

### 2.2 Metabolic Rewiring of HCC Cells in Response to Glutamine Depletion

**Figure 2:**
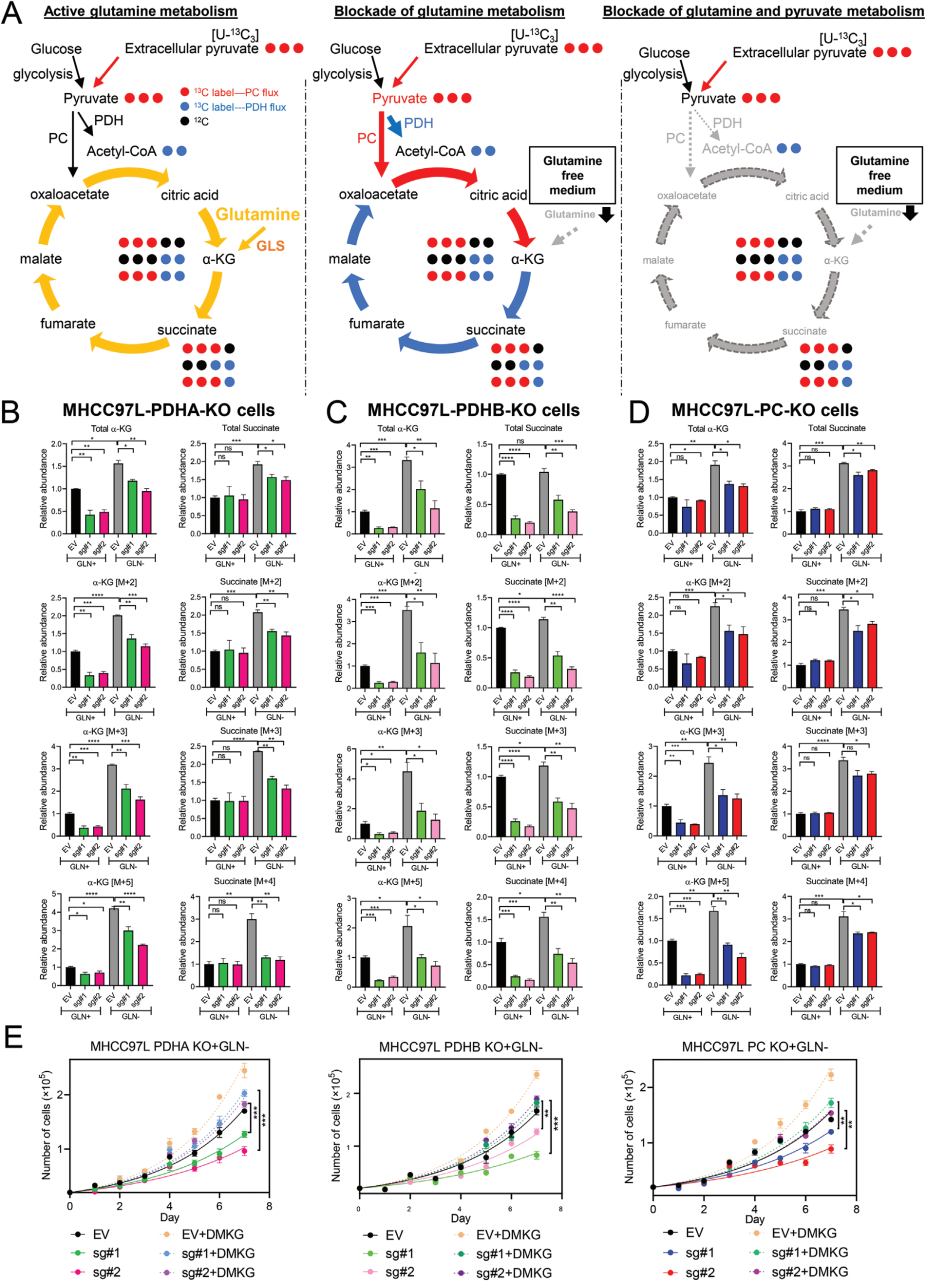
Metabolic characteristics of HCC cells in response to glutamine depletion. A) TCA cycle flux under glutamine supplementation or glutamine depletion. B–D) Stable‐isotope tracing with [U‐^13^C_3_] pyruvate revealed that B) PDHA, C) PDHB, or D) PC KO slowed TCA flux by decreasing the production of *α*‐ketoglutarate (*α*‐KG) and succinate. E) Cell proliferation assays demonstrated that the addition of dimethyl ketoglutarate (DMKG) rescued the proliferation of PDHA, PDHB, and PC KO cells under glutamine depletion (GLN−). *p < 0.05, **p < 0.01, ***p < 0.001, ****p < 0.0001 versus EV as indicated. Student's *t*‐test. Error bars indicate mean ± SEM (*n* = 3).

To understand the role of pyruvate metabolism in the metabolic adaptation of HCC cells under glutamine depletion, we performed stable‐isotope tracing studies with uniformly labelled [U‐^13^C_3_] sodium pyruvate in PDHA, PC and PDHB KO clones by gas chromatography‐mass spectrometry (GC‐MS) analysis. Metabolic flux analysis revealed the increasing accumulations of isotopologue metabolites in TCA cycle in MHCC97L‐EV (empty vector, EV) group when glutamine is depleted, suggesting an increased reliance on pyruvate (**Figure** **2**A–D). Restriction of pyruvate metabolism through PDH flux (KO of PDHA or PDHB), which limited the conversion of pyruvate into acetyl‐CoA (two carbon unit) leading to reduction flow of two carbon units to TCA cycle (labelled as blue dots in Figure 2A) or restriction of PC flux (KO of PC) that inhibited the conversion of pyruvate into oxaloacetate leading to reduction flux of three carbon labelled units (labelled with red dots in Figure 2A), decelerated the flux in TCA cycle (Figure 2A–D). Under glutamine depletion, KO of PDHA or PDHB dramatically decreased the production of two carbon labelled (M+2) isotopologues of *α*‐KG and succinate (Figure 2B,C), indicating the restriction of flux through PDH. KO of PC decreased the production of three carbon labelled (M+3) isotopologues *α*‐KG and succinate under glutamine depletion (Figure 2D), indicating the restriction of flux through PC. Interestingly, KO of either PDHA or PDHB indirectly reduced the activity of PC as indicated by the reduced representative metabolites through PC flux (labelled as red dots in Figure 2A), M+3 and M+5 isotopologues of *α*‐KG and M+3 and M+4 isotopologues of succinate under glutamine depletion (Figure 2B,C). Reversely, KO of PC also indirectly reduced the activity of PDH as indicated by the reduced representative metabolites through PDH flux (labelled as blue dots in Figure 2A), M+2 and M+5 isotopologues of *α*‐KG and M+2 and M+4 isotopologues of succinate under glutamine depletion (Figure 2D). Therefore, under glutamine depletion, TCA cycle was replenished by extracellular pyruvate directing through PDH and PC to support HCC cell survival and proliferation. KO of either PDH or PC could restrict the flux of pyruvate metabolism into TCA cycle. Carbon tracing results revealed that the significant reduction of *α*‐KG in PDHA KO, PDHB KO and PC KO clones after glutamine depletion, suggesting that *α*‐KG may be a critical intermediate for glutamine to replenish the TCA cycle for anaplerosis. The oxidation of *α*‐KG in the TCA cycle produces succinate and fumarate to support HCC cell proliferation and biosynthesis. In addition, *α*‐KG also serves as an important substrate for histone modification during cancer development. The addition of soluble form of *α*‐KG (Dimethyl‐α‐ketoglutarate, DMKG [4 mm]) could restore the viability of PDHA, PDHB and PC KO clones under glutamine depletion (GLN−, 0 mm) (Figure 2E), suggesting that the effects of glutamine and pyruvate metabolism inhibition on HCC are at least partially caused by *α*‐KG.

### 2.3 KO of PDH or PC Disrupted Mitochondrial Functions in HCC Cells Under Glutamine Depletion

**Figure 3:**
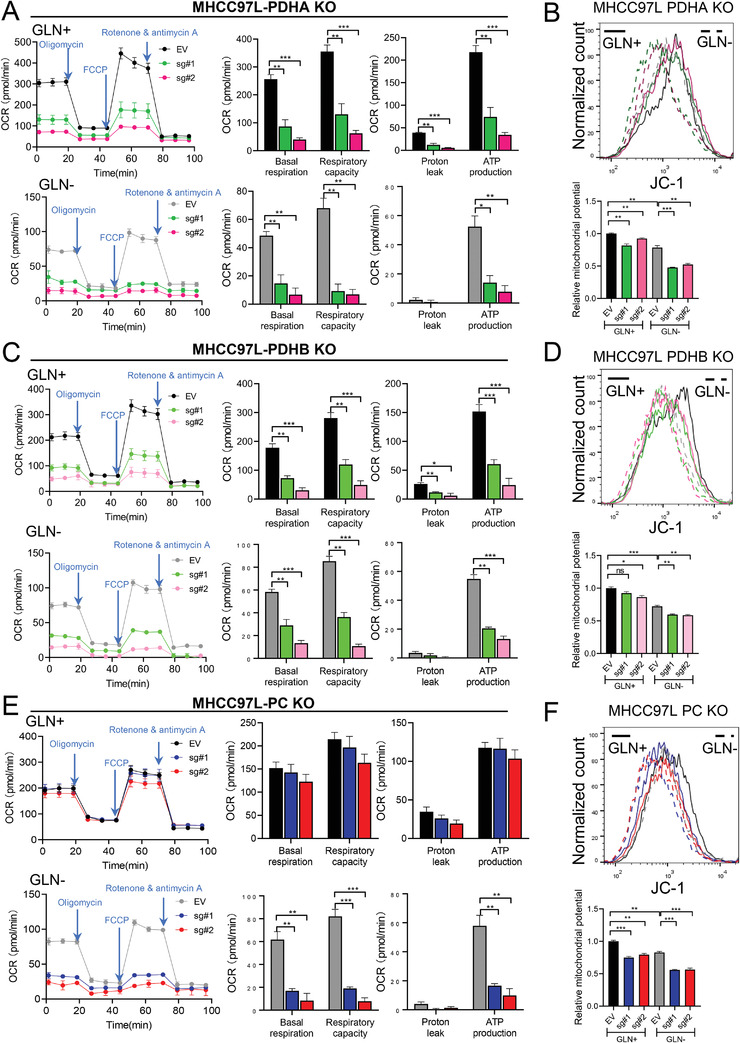
Mitochondrial function affected by knockout of PDH and PC in HCC cells under glutamine depletion. A,C,E) Seahorse XF Cell Mito Stress assay demonstrated that KO of A) PDHA, C) PDHB, and E) PC significantly suppressed oxygen consumption rate (OCR) of MHCC97L cells under glutamine depletion (GLN−) compared to normal conditions with glutamine (GLN+). B,D,F) JC‐1 staining in B) PDHA, D) PDHB, and F) PC KO cells revealed significantly impaired mitochondrial membrane potential of MHCC97L cells under glutamine depletion (GLN−) compared to normal conditions with glutamine (GLN+). GLSi (BPTES): 1 µm. *p < 0.05, **p < 0.01, ***p < 0.001, ****p < 0.0001 versus EV as indicated. Student's *t*‐test. Error bars indicate mean ± SEM (*n* = 3).

TCA cycle is important for mitochondrial respiration. We next investigated the effects of glutamine deficiency and blockade of pyruvate metabolism on the mitochondrial functions of HCC cells. Seahorse XF Cell Mito Stress Test indicated that KO of PDHA decreased the oxygen consumption rates (OCRs) and respiratory capacity in HCC cells (**Figure** **3**A). Near‐complete inhibition of mitochondrial activity was observed in PDHA, PDHB, and PC KO HCC cells under glutamine depletion (Figure 3A,C,E). Consistently, JC‐1 staining which indicate mitochondrial membrane potential was decreased in PDHA, PDHB and PC KO HCC cells especially under glutamine depletion, suggesting that the mitochondria were depolarized with decreased activity (Figure 3B,D,F).

PDH inhibitor CPI‐613 has been approved by FDA for clinical trials for pancreatic cancer treatment.^[^ ^23^ ^]^ Previous study also indicated that the PC inhibitor CHCA (*α*‐cyano‐4‐hydroxycinnamic acid) effectively suppressed breast cancer in vivo.^[^ ^24^ ^]^ Similar results could be observed when MHCC97L cells were treated with PDH or PC inhibitor and glutaminase (GLS) inhibitor BPTES (Figure S3A,B, Supporting Information). Of note, both OCR and mitochondrial membrane potential measurements showed that the mitochondrial activity of HCC cells was generally decreased when glutamine was withdrawn. This is reasonable as the depletion of glutamine reduced *α*‐KG which led to the failure of TCA cycle replenishment, thereby indirectly dampening the mitochondrial activity. These data demonstrated that HCC cells greatly relied on pyruvate metabolism to maintain mitochondrial activity especially in glutamine depleted condition.

### 2.4 PDH and PC Inhibitors Sensitized HCC Cells to Glutamine Deficiency

**Figure 4:**
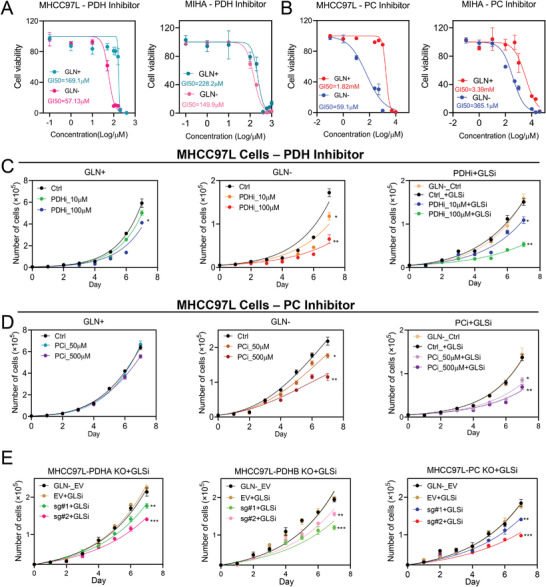
The effects of PDH, PC, and GLS inhibitors on HCC cells. A,B) Higher GI_50_ for A) PDH and B) PC inhibitors were detected on MIHA compared to MHCC97L cells using XTT assay. C,D) Cell proliferation assay demonstrated C) PDH and D) PC inhibitor significantly suppressed MHCC97L cell proliferation in dose‐dependent manner under glutamine depletion (GLN−) or in combination with GLS inhibitor (GLSi) treatment. E) Proliferation of MHCC97L‐PDHA, ‐PDHB, and ‐PC KO cells in the presence of GLS inhibitor (GLSi) were significantly suppressed. GLSi (BPTES): 1 µm, *p < 0.05, **p < 0.01, ***p < 0.001, ****p < 0.0001 versus Ctrl or EV as indicated. Student's *t*‐test. Error bars indicate mean ± SEM (*n* = 3).

Next, we determined the GI_50_ of PDH inhibitor (CPI‐613) and PC inhibitor (CHCA) in MHCC97L cells and normal hepatocytes MIHA, respectively (**Figure** **4**A,B). The GI_50_ of both PDH and PC inhibitors were lower in MHCC97L cells than MIHA (Figure 4A,B), suggesting the inhibitory doses for PDH and PC inhibitors on HCC cells may not affect normal hepatocytes. More interestingly, we found that the GI_50_ of PDH and PC inhibitors were much lower in HCC cells under glutamine depletion (GLN−) (Figure 4A,B). In addition, both PDH and PC inhibitors remarkably suppressed HCC cell proliferation in glutamine depleted condition (GLN−) (Figure 4C,D). The treatments did not cause cell death under glutamine replete condition (GLN+), while an increase of cell death was detected after PDH/PC inhibitor treatment under glutamine depletion (Figure S4A–D, Supporting Information).

**Figure 5:**
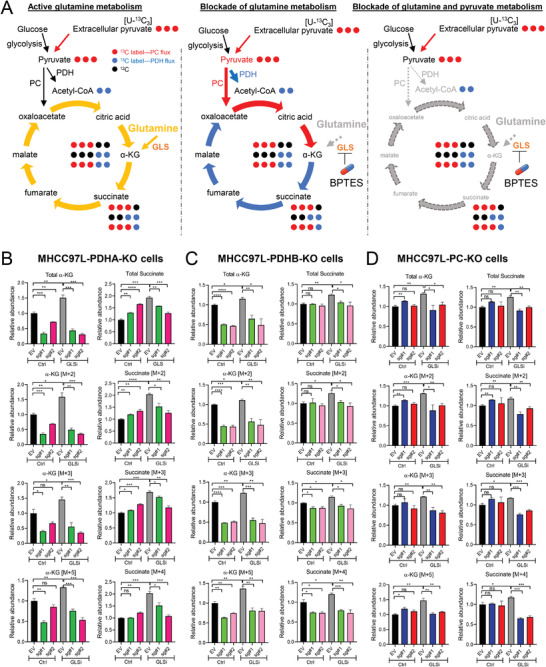
Metabolic flux of HCC cells in response to glutaminase (GLS) inhibition (BPTES) tracing with [U‐^13^C_3_] pyruvate. A) TCA cycle flux under glutamine supplementation with or without GLS inhibitor (BPTES). BPTES: 1 µm. B–D) Stable isotopic carbon tracing using [U‐^13^C_3_] pyruvate revealed that B) PDHA, C) PDHB, or D) PC KO slowed down TCA flux by decreasing the production of *α*‐ketoglutarate (*α*‐KG) and succinate. *p < 0.05, **p < 0.01, ***p < 0.001, ****p < 0.0001 versus EV as indicated. Student's *t*‐test. Error bars indicate mean ± SEM (*n* = 3).

Glutaminase (GLS) is the first enzyme that metabolizes glutamine to generate glutamate, a precursor of *α*‐KG which enters the TCA cycle. GLS has been considered as an attractive molecular target in glutamine metabolism in cancer treatment and tumor therapy. As proof‐of‐concept, we also asked whether pharmacologic inhibition of GLS resembles glutamine depletion in metabolic rewiring and repressing the growth of HCC. BPTES, the GLS inhibitor, has been widely applied to suppress the conversion of glutamine to glutamate in cancer study.^[^ ^25^, ^26^ ^]^ Interestingly, we found the combined treatment of PDH or PC inhibitor and BPTES most significantly suppressed the proliferation of HCC cells than single treatments of PDH/PC inhibitor or GLS inhibitor. Consistently, PDHA, PDHB, or PC KO also sensitized HCC cells to BPTES treatment (Figure 4C,D). Next, we performed stable isotopic carbon tracing using [U‐^13^C_3_]‐sodium pyruvate in PDHA, PDHB, and PC KO cells treated with GLS inhibitor (BPTES) (**Figure** **5**). Consistent with glutamine depletion, GLS inhibition increased the pyruvate flux through PDH and PC in HCC cells (EV) (Figure 5A–D), whereas KO of PDHA, PDHB, or PC decelerated the flux through PDH, or PC as indicated by the reduced [M+2] or [M+3] labelled *α*‐KG and succinate (Figure 5B–D).

### 2.5 PDH or PC Inhibitor Suppressed the Growth of HCC In Vivo

**Figure 6:**
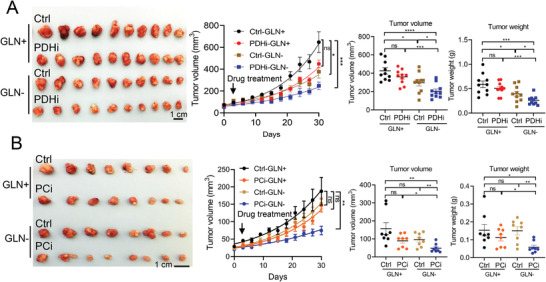
PDH inhibitor (PDHi) or PC inhibitor (PCi) sensitized HCC to glutamine deficient diet. A) Tumor images and tumor volume growth curves after treated with PDH inhibitor (PDHi; CPI‐613, 4 mg kg^−1^) and normal, glutamine supplemented (GLN+) or glutamine deficient (GLN−) diets. Ctrl/GLN+: control with normal diet; PDHi/GLN+: CPI‐613 treatment with normal diet; Ctrl/GLN−: control with glutamine deficient diet; PDHi/GLN−: CPI‐613 treatment with glutamine deficient diet; tumor volumes and tumor weights of mice in treated groups. (*n* = 10). B) Tumor images and tumor volume growth curves after treated with PC inhibitor (PCi; CHCA, 40 mg kg^−1^) and normal, glutamine supplemented (GLN+) or glutamine deficient (GLN−) diets. Ctrl/GLN+: control with normal diet; PCi/GLN+: CHCA treatment with normal diet; Ctrl/GLN−: control with glutamine deficient diet; PCi/GLN−: CHCA treatment with glutamine deficient diet; tumor volumes and tumor weights of mice in treated groups (*n* = 8). *p < 0.05, **p < 0.01, ***p < 0.001, ****p < 0.0001 versus Ctrl or as indicated. Student's *t*‐test. Error bars indicate mean ± SEM.

Next, we asked whether dietary intervention of glutamine depletion could be employed in combination with PDH or PC inhibitor as HCC treatment. We investigated whether PDH inhibitor (PDHi, CPI‐613) or PC inhibitor (PCi, CHCA) could suppress HCC more effectively under a glutamine deficient diet than normal diet. Nude mice were inoculated with MHCC97L cells subcutaneously. After the tumors were palpable, mice were randomized into four groups and pre‐treated with the diet (glutamine supplemented diet or glutamine deficient diet) for 3 days before the administration of PDHi or PCi intraperitoneally (Figure S5A, Supporting Information). Consistent with Figure 1A, we found that glutamine deficient diet repressed tumor growth as compared to glutamine supplemented diet (normal diet, GLN+). Single treatment of PDH or PC inhibitor slightly suppressed the tumor growth (**Figure** **6**A,B). Strikingly, PDH or PC inhibitor combined with glutamine deficient diet (GLN−) demonstrated the greatest tumor suppressive effects than single treatments or glutamine deficient diet alone in vivo, as tumor volume and weights were significantly lower (Figure 6A,B) without significant body weight loss (Figure S5B,C, Supporting Information). To verify our results with pharmacologic blockade of glutamine, we tested the efficacy of GLS inhibitor (GLSi, BPTES) in combination with PDH inhibitor (PDHi, CPI‐613) or PC inhibitor (PCi, CHCA) in mice with subcutaneous tumors derived from MHCC97L. Single treatment of either PDHi, PCi, or GLSi slightly inhibited the tumor growth (Figure S6A,B, Supporting Information). The combined treatment (combo) of PDHi or PCi with GLSi restrained HCC growth is the most as compared to control and single treatments without affecting the body weights (Figure S6A,B, Supporting Information).

**Figure 7:**
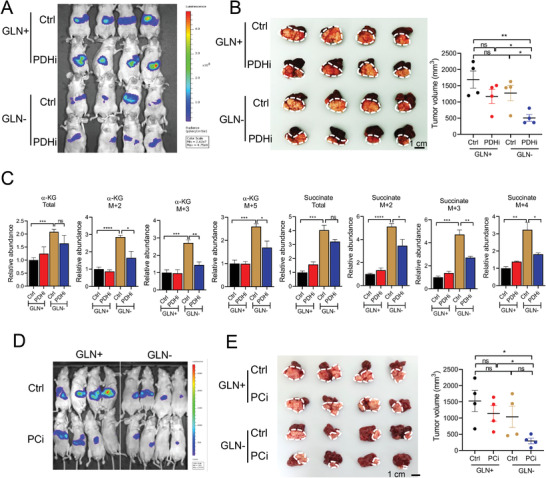
Efficacy of glutamine deficient diet combined with PDH or PC inhibitor in orthotopic HCC model. A) Bioluminescent image of orthotopic tumors implanted in mice treated with Ctrl or PDH inhibitor (PDHi, CPI‐613, 4 mg kg^−1^) and glutamine supplemented or deficient (GLN+/−) diets. B) Representative image of the orthotopic tumors and tumor volumes. C) Labelled metabolites detected by in vivo carbon tracing with U‐^13^C_6_‐glucose. D) Bioluminescent image of orthotopic tumors implanted in mice treated with Ctrl or PC inhibitor (PCi; CHCA, 40 mg kg^−1^) and glutamine supplemented or deficient (GLN+/−) diets. E) Representative image of the orthotopic tumors and tumor volumes. *p < 0.05, **p < 0.01, ***p < 0.001, ****p < 0.0001 versus Ctrl or as indicated. Student's *t*‐test. Error bars indicate mean ± SEM.

To further confirm the effects of our treatments in tumors in native liver microenvironment, we performed orthotopic implantation with MHCC97L cells in nude mice. Combination of PDH or PC inhibitor with glutamine deficient diet showed significantly greater suppressive effects on HCC than single treatments (**Figure** **7**A,B,D,E) with no adverse effects on mice as reflected by the unchanged body weights (Figure S7, Supporting Information). To confirm the metabolic rewiring in orthotopic HCC tumors, we performed in vivo carbon tracing with [U‐^13^C_6_] glucose in mice. We delivered [U‐^13^C_6_] glucose intravenously in mice 60 min before we sacrificed the mice to study the metabolic flux in HCC. We found that the treatment of glutamine deficient diet diverted more ^13^C‐glucose into TCA cycle as shown by the increased ^13^C‐labelled TCA metabolites, *α*‐KG, and succinate (Figure 7C). However, PDH inhibitor treatment abolished the fueling of glucose to TCA cycle accompanying the reduction of ^13^C‐labelled TCA metabolites (Figure 7C). The mass spectrometry analysis further confirmed the metabolic reprogramming in tumor microenvironment induced by glutamine deficiency. We also demonstrated that targeting pyruvate metabolism could overcome the potential metabolic adaptation caused by glutamine depletion, highlighting the potential of combined treatment involving dietary intervention to withdraw glutamine and PDH inhibitor or PC inhibitor.

## 3 Discussion

Increasing evidence suggested that diet is associated with cancer. Epidemiology data indicated that dietary reduction of carbohydrate intake could decrease the risk of ER‐positive breast cancer.^[^ ^27^ ^]^ Ketogenic diets were also shown to improve the clinical outcome for breast cancer patients.^[^ ^28^ ^]^ Recently, a number of reports have demonstrated that dietary interventions could effectively affect tumor development and drug response in mouse cancer models. Caloric restriction or low glycemic diet has been shown to be able to repress pancreatic cancer growth through repressing stearoyl‐CoA desaturase (SCD), leading to reduction of monosaturated fatty acids.^[^ ^29^ ^]^ Dietary methionine restriction also repressed colorectal cancer through modulating the metabolites in one carbon metabolism which supports antioxidant and nucleotide synthesis. Dietary methionine restriction synergized with radiation or chemotherapy, 5‐fluorouracil (5‐FU, a thymidylate synthase inhibitor) to suppress colorectal cancer and soft‐tissue sarcoma growth in mice.^[^ ^30^ ^]^ Interestingly, the mouse experimental model showed that dietary methionine restriction prior or after cancer cell inoculation could prevent and suppress tumor growth suggesting that dietary methionine promotes both the onset and development of colorectal cancer.^[^ ^30^ ^]^ Histidine supplementation was able to sensitize tumors to methotrexate treatment as the supplementation promotes histidine catabolism which consumes tetrahydrofolate, a limiting metabolite that determines the sensitivity of the leukemia cancer cells to methotrexate.^[^ ^31^ ^]^ On the other hand, dietary fructose was able to promote ketohexokinase (KHK)‐dependent conversion of fructose‐1‐phosphate which enhanced glycolysis and de novo lipogenic pathway in intestinal cancer.^[^ ^32^ ^]^ These findings showed that cancer growth can be greatly affected by diets. Dietary depletion of specific amino acid as a therapeutic approach has never been tested in liver cancer. To our knowledge, our study is the first to employ dietary glutamine restriction as a treatment for liver cancer. Our CRISPR‐Cas9 genome‐wide library screening further identified the potential adaptive mechanism through enhancing pyruvate metabolism during glutamine depletion to enhance the efficiency of this new treatment regimen.

Interestingly, previous CRISPR library screening studies indicated that HCC cells adapt by rewiring their metabolic machinery under different nutrient‐limited conditions. Niu et al. found that aldolase A was induced by hypoxia‐inducible factor *α* and epigenetic modifications to overcome hypoxia in HCC cells.^[^ ^33^ ^]^ Another study suggested that adenylosuccinate lyase (ADSL) is the key enzyme involved in purine metabolism and mitochondrial function during the formation of liver cancer.^[^ ^34^ ^]^ Liver cancer cells like many other cancer cells are addicted to glutamine. A chemical library screening of 13 compounds identified glutamine transporter inhibitor targeting alanine‐serine‐cysteine transporter, type‐2 (ASCT2, encoded by gene SLC1A5), V‐9302, showed the greatest synergism with GLS inhibitor, CB‐839, in suppressing HCC cells. Mechanistically, upon blockade of GLS, HCC cells were shown to alternatively take up glutamine through ASCT2 to generate glutathione which counteracted ROS; therefore, co‐blockade of GLS and ASCT2 induced ROS, limiting HCC growth.^[^ ^35^ ^]^ Here, we further showed that glutamine deficient diet significantly suppressed HCC in multiple mouse HCC models. Using metabolic tracing, we confirmed that upon glutamine depletion, liver cancer cells rewired metabolically to consume extracellular pyruvate to replenish the TCA cycle. As proof‐of‐concept, we demonstrated that dietary glutamine restriction in combination with pharmacological inhibitors targeting PDH or PC most effectively suppressed HCC than single treatments. In addition, we found that GLS inhibitor resonated the effects of glutamine deficient diet as single or combined treatment with PDH or PC inhibitor. Although adverse side effects in vivo were not found in both glutamine deficient diet and GLS inhibitor during the experiments, we believe that a glutamine deficient diet might be less toxic than GLS inhibitors due to possible off‐target effects of small molecule inhibitors which might hit other important enzymes in normal cells. Furthermore, variations in different animal experiments can be caused by different factors such as the conditions of experimental mice (age and body weights), HCC cells, and source of the Matrigel.

The tumor microenvironment of liver cancer consists of malignant and a variety of immune cells.^[^ ^36^, ^37^, ^38^ ^]^ Generally, cancer and actively proliferating T cells share common metabolic machinery and nutrient requirements. Both cancer and proliferating T cells are dependent on glucose and glycolysis to maximize the output from anabolic reactions.^[^ ^39^ ^]^ Interestingly, glutamine antagonist 6‐diazo‐5‐oxo‐L‐norleucine (DON) prodrug, JHU083, showed remarkable effects in blocking the TCA cycle and growth of colorectal cancer. However, JHU083 did not dampen the anti‐tumor effect of T cells but favored tumor infiltrated CD8^+^ T cell proliferation, activation, and longevity. T cells adapted to glutamine blockade by increasing acetate flux to replenish the TCA cycle in colorectal cancer.^[^ ^40^ ^]^ Although the general anabolic requirements of actively dividing cancer and immune cells are similar, their speed and abilities in adapting the blockade of specific metabolic pathways are unique. Based on the finding from C. H. Chang et al., it is expected that glutamine deficient diet should not inhibit T cells, highlighting its potential to be used in combination with existing FDA‐approved immunotherapies for HCC. On the other hand, knockout of either PDH or PC would affect the activity of the other one and slow down the flux of pyruvate metabolism fueling TCA cycle. The specificities of the knockout cell lines were verified by western blotting. Future studies should also focus on the characterization of the metabolic programs of various cell types within the liver cancer microenvironment to facilitate the design of therapeutic approaches that specifically perturb metabolic program of cancer but not anti‐tumor immune cells.

## 4 Conclusion

In summary, our study showed that dietary restriction of glutamine could be employed as a therapeutic regimen for HCC. Our functional screening identified the metabolic adaptability of HCC cells toward glutamine withdrawal through induction of pyruvate metabolism. ^13^C metabolic flux study showed that HCC cells exhibited increased pyruvate uptake and enhanced PDH and PC activities to convert pyruvate into oxaloacetate and acetyl‐CoA which are precursors of citric acid to initiate the TCA cycle. Meanwhile, we showed that possible metabolic compensation through pyruvate metabolism upon glutamine withdrawal could be overcome by pharmacological inhibitors against PDH and PC. Our study exemplified the therapeutic potential of dietary intervention as part of liver cancer treatment. Metabolic inhibitors are one of the common classes of chemotherapies used in cancer treatment. As the metabolic features of cancer cells are revealed in the last decade due to the advancement of mass spectrometry methods, future studies are needed to examine the efficiency of dietary depletion of different cancer specific metabolites. Deepened knowledge in this area will make great leap toward the safer design of anti‐cancer therapeutic regimens.

## 5 Experimental Section

### Genome‐Wide CRISPR/Cas9 Knockout Library Screening

A genome‐wide CRISPR/Cas9 KO screening was performed to systematically evaluate the underpinning adaptive mechanisms of glutamine depletion in human HCC cells. MHCC97L was a gift from Dr. Z.Y. Yang (Fudan University of Shanghai). MHCC97L cells which stably expressed Cas9 were generated by the lentiviral transduction of Cas9 coding sequence. The Human GeCKO v2A CRISPR‐Cas9 knockout pooled library (Addgene, Watertown, MA, USA) was a gift from Prof. Feng Zhang (Board Institute). A human GeCKO v2 CRISPR library A was stably transfected into MHCC97L‐Cas9 cells. The transduced cells were selected with 1.6 µg mL^−1^ of puromycin for 7 days to generate the mutant cell pool. Mutant MHCC97L cells were then cultured in medium with (4 mm; GLN+) or without (0 mm; GLN−) glutamine for 7 days, respectively. After treatment, cells were harvested for genomic DNA (gDNA) extraction and sgRNA sequences were amplified using NEB Next High‐Fidelity 2× PCR Master Mix and subjected to massive parallel amplicon sequencing carried out by Novogene Technology (Beijing, China). The sgRNA read count and hits calling were analyzed by MAGeCK v0.5.7 algorithm.^[^ ^41^ ^]^

### Establishment of PDHA/PDHB/PC Knockout HCC Cell Lines

MHCC97L‐Cas9 stable cells were established by lentiviral transduction with lentiCas9‐Blast vector (Addgene). Cas9 protein expression was confirmed by western blotting using a Cas9‐specific antibody (Cell Signaling Technology, Danvers, MA, USA; #14697, dilution 1:1000). PDHA, PDHB, and PC‐KO cell lines were established by CRISPR‐Cas9 KO using MHCC97L‐Cas9 cells with pLentiGuide‐Puro vector (Addgene). pLentiGuide‐Puro vectors were introduced into MHCC97L‐Cas9 cells through lentiviral transduction and cells infected with pLentiGuide‐Puro vectors were selected by 1 µg mL^−1^ puromycin (Sigma‐Aldrich, St. Louis, MO, USA). The sgRNA sequences are listed in Table S1, Supporting Information.

### Western Blotting

Total protein lysate was extracted by radioimmunoprecipitation assay (RIPA) lysis buffer with cOmplete protease inhibitor and PhosSTOP phosphatase inhibitor cocktails (both from Roche, Basel, Switzerland). PDHA, PDHB, PC, and *β*‐actin were separated by 10% v/v sodium dodecyl sulfate‐polyacrylamide gel electrophoresis (SDS‐PAGE) and transferred onto PVDF membranes (GE Healthcare, Chicago, IL, USA). The protein expression of PDHA, PDHB and PC in different subclones was determined by western blotting using anti‐PDHA (Cell Signaling, #3205, 1:1000), anti‐PDHB (Abcam, Cambridge, UK; ab155996, 1:1000) and anti‐PC (Santa Cruz Biotechnology, Dallas, TX, USA; sc271493, 1:1000) antibodies, respectively.

### Cell Proliferation

HCC cells were seeded onto 12‐well plates at a density of 2 × 10^4^ cells/well in triplicates and maintained in a humidified incubator at 37 °C with CO_2_. Culture media with glutamine supplementation and/or glutamine depletion (or inhibitor or vehicle control) were refreshed every other day. Cell numbers were counted by TC20 Automated Cell Counter (Bio‐Rad Laboratories, Hercules, CA, USA).

### Animal Experiments

For HDTVi model, genome‐editing plasmid DNA mixtures containing Sleeping Beauty (SB) transposon system over‐expressing c‐Myc (c‐Myc^OE^) and CRISPR‐Cas9 system knocking out Tp53 (Tp53^KO^) in saline at a volume equivalent to 10% body weight were injected into the tail vein of 6–8 weeks old male C57BL/6N mice within 6–8 s as previously described.^[^ ^20^ ^]^ Two weeks after injection, mice were randomly separated into two groups, one group was fed with normal diet (with glutamine supplementation, GLN+) while the other was fed with glutamine deficient diet (GLN−). GLN+ diet (A10021B, glutamine: 5% [g, w/w]; glutamate: 30% [g, w/w]) and GLN− diet (A20081701, glutamine: 0% [g, w/w]; glutamate: 0% [g, w/w]) were both purchased from Research Diets, Inc. (New Brunswick, NJ, USA). The body weights of all mice were recorded throughout the experiments.

For subcutaneous implantation model, 2 × 10^6^ MHCC97L cells were resuspended in 50 µL PBS and mixed with 50 µL Matrigel (v:v = 1:1) (BD Biosciences, Franklin Lakes, NJ, USA) on ice and were subcutaneously injected into flanks of 6‐ to 8‐week‐old male BALB/cAnN‐nu (nude) mice. Tumor size was measured with an electronic caliper and tumor volume was calculated using the following formula:

(1) $$\begin{array}{ccc} \mathrm{Tumor}\;\mathrm{volume}\;({\mathrm{mm}}^{3}) & = & 0.52\times \mathrm{length}\left(\mathrm{mm}\right)\times \mathrm{width}\left(\mathrm{mm}\right)\\ & & \times \;\mathrm{height}\left(\mathrm{mm}\right) \end{array}$$

For orthotopic tumor implantation model, 1.5 × 10^6^ luciferase‐labeled MHCC97L cells were resuspended in 100% Matrigel (BD Biosciences) on ice and injected into the left lobes of the livers of 6‐ to 8‐week‐old male nude mice. Two weeks and six weeks after implantation, mice were administered 100 mg kg^−1^ D‐luciferin (PerkinElmer, Waltham, MA, USA) via intraperitoneal injection and underwent bioluminescent imaging using the Xenogen IVIS 100 Imaging System (Caliper, Hopkinton, MA, USA) to examine tumor size. The lungs of the mice were harvested for ex vivo imaging.

Drug administration began 2 weeks post‐injection when tumors were palpable. The mice were randomized into four groups and treated with: a) control (saline); b) PDH inhibitor (PDHi) (CPI‐613: 4 mg kg^−1^ per 6 days) alone or PC inhibitor (PCi) (CHCA: 40 mg kg^−1^ per day) alone; c) GLS inhibitor (GLSi) (BPTES: 10 mg kg^−1^ per 3 days) alone; and d) combined treatment of PDHi and GLSi or cotreatment of PCi and GLSi.

Dietary intervention began 2 weeks after tumor implantation in HCC tumor‐bearing mice. Mice were randomized into four groups and pre‐fed with either normal diet (GLN+) or glutamine deficient diet (GLN−) (Research Diets) as mentioned for 3 days. Then, mice were administrated with either PDHi or PCi as follows: a) saline and GLN+ diet; b) PDHi (CPI‐613: 4 mg kg^−1^ per 6 days) or PCi (CHCA: 40 mg kg^−1^ per day) and GLN+ diet; c) saline and GLN− diet; and d) PDHi (CPI‐613: 4 mg kg^−1^ per 6 days) or PCi (CHCA: 40 mg kg^−1^ per day) and GLN− diet.

All experimental procedures were performed according to the Animals (Control of Experiments) Ordinance of Hong Kong. All animal experiments were approved (CULATR 5826‐21) by the Committee on the Use of Live Animals in Teaching and Research, The University of Hong Kong.

### Stable Isotope Carbon Tracing Experiments

For in vivo tracing, HCC‐bearing mice were administered single injection of [U‐^13^C_6_] glucose (1.6 mg g^−1^ body weight) through the tail vain (i.v.) as reported.^[^ ^42^ ^]^ Mice were sacrificed 60 min post‐ injection and tumor tissues were rapidly harvested, snap‐frozen with liquid nitrogen, and stored at −80 °C prior to metabolite extraction. Metabolites were extracted from tissues weighing between 50–100 mg in a methanol:water (80:20, v/v) extraction solution after homogenization. The extracted solution was centrifuged at 4 °C at 13 000 rpm for 15 min, and the supernatants were transferred to new tubes. The supernatants were dried with a concentrator and stored at −80 °C for further analysis.

For in vitro tracing experiments, the PDHA, PDHB and PC KO cells were seeded onto 60 mm dishes in triplicates and treated with tracing medium (DMEM high glucose medium added with U‐^13^C_3_ sodium pyruvate [1 mm]) supplemented with glutamine (GLN+, 4 mm) or without glutamine (GLN−, 0 mm) for 48 h. For experiments involving GLS inhibitor, the PDHA, PDHB, and PC KO cells were seeded onto 60 mm dishes in triplicates and treated with tracing medium (DMEM high glucose medium added with U‐^13^C_3_ sodium pyruvate [1 mm]) supplemented with glutamine (GLN+, 4 mm) with 1 µm GLS inhibitor (BPTES) or control for 48 h. Media were withdrawn from dishes and washed with PBS. Ice‐cold 80% methanol (methanol:water, v:v, 80:20) was added directly to the dishes and cells were scraped and transferred to Eppendorf tubes followed by 3 freeze–thaw cycles in liquid nitrogen. The extracted samples were centrifuged at 4 °C at 13 000 rpm for 15 min and the supernatants were transferred to new tubes. The supernatants were dried with a concentrator and stored at −80 °C for further analysis.

The dried residue was derivatized as previously reported.^[^ ^43^ ^]^ Briefly, the dried samples were initially resolved with 30 µL of methoxyamine (solution of methoxyamine hydrochloride in pyridine, 20 mg mL^−1^) containing internal standard (ribitol, 2 µg mL^−1^) and incubated at 37 °C for 1 h. Then, each sample was added with 35 µL of BSTFA plus 1% of TMCS (B‐023, Sigma), incubated at 65 °C for 90 min, and cooled at room temperature. The samples were transferred to glass inserts immediately for subsequent analysis by GC‐MS within 24 h. The metabolites in TCA cycle were analyzed with an Agilent GC‐MS system consisting of an Agilent 7890B GC coupled to Agilent 5975C mass selective detector. The GC was operated in splitless mode with constant helium gas flow at 1 mL min^−1^. The derivatized samples were injected into an DB‐5MS column (Agilent Technologies, Santa Clara, CA, USA; #122‐5532) and the GC oven program was set as: started at 80 °C and held for 1 min, then increased by 10 °C min^−1^ to 320 °C, held at 320 °C for 5 min. Peaks representing compounds of interest were extracted and integrated using MassHunter software (Agilent).

### Oxygen Consumption Rates (OCRs) Measurement

OCRs of PDHA, PDHB, and PC KO subclones were measured by using XFp Cell Mito Stress Test (Agilent) according to manufacturer's protocol. HCC KO subclones were seeded onto miniplates with a density of 1 × 10^4^ cells per well and maintained with glutamine supplementation (GLN+, 4 mm) or glutamine depletion (GLN−, 0 mm) medium for 48 h. The cells were washed with assay medium (pH = 7.0) and incubated in a non‐CO_2_ incubator for 1 h before analysis. 1 µm oligomycin (complex V inhibitor), 0.5 µm FCCP (disrupts mitochondrial membrane potential), and 0.5 µm rotenone/antimycin A (complex I and III inhibitors) mixture were sequentially injected into the wells at indicated time points prior to OCR measurements by the Seahorse XFp Analyzer (Agilent). OCR was quantified according to the mitochondrial function profile determined by basal respiration, ATP‐linked respiration, proton leak, and maximal respiration which were calculated by the Seahorse XF Report Generator (Agilent).

### Mitochondrial Membrane Potential Measurements

MHCC97L cells were seeded onto 6‐well plates at a density of 2 × 10^5^ cells per well and maintained in glutamine supplementation (GLN+, 4 mm) or glutamine depletion (GLN−, 0 mm) medium for 48 h. For mitochondrial membrane potential measurement, cells were stained with 5,5′,6,6′‐tetrachloro‐1,1′,3,3′‐tetraethyl‐benzimidazolylcarbocyanine chloride (JC‐1) (Invitrogen, Waltham, MA, USA) at a final concentration of 2 µm for 15 min at 37 °C. Cells were directly scraped and resuspended in 0.5 mL PBS for flow cytometry analysis.

### Cell Viability Assays

Cell viability was determined with Cell Proliferation Kit II (XTT) (Roche). XTT labeling master mix was prepared by mixing an XTT labeling reagent and electron coupling regent at a ratio of 50:1. 50 µL XTT labeling master mix was then added to each well and the cells were incubated for 2 h at 37 °C. Absorbance was measured at 450 nm by Infinite F200 plate reader (Tecan, Männedorf, Switzerland).

### Statistical Analysis

For carbon tracing, the in vitro tracing data were normalized to cell number and internal standard and the in vivo tracing data were normalized to tissue weight and internal standard. All statistical analyses were performed using GraphPad Prism 8.0 software (GraphPad Software, Inc., San Diego, CA, USA). Data are representative of at least three independent experiments and are expressed as mean ± SEM. The detailed sample size (*n*) for each statistical analysis was indicated in individual figure legend. Comparisons between two independent groups were assessed by two‐tail Student's *t*‐test. Differences between values were considered statistically significant when *p < 0.05, **p < 0.01, ***p < 0.001, ****p < 0.0001.

## Conflict of Interest

The authors declare no conflict of interest.

## Author Contributions

The concept and design of this study: C.Y. and C.C.L.W. Performed experiments: C.Y., D.L., M.S.Z., A.P.W.T., L.W., M.H.R.B., B.P.Y.W., C.Y.K.C., V.W.H.Y., and Y.C. Analysis and interpretation of data: C.Y., D.L., M.S.Z., A.P.W.T., L.W., M.H.R.B., B.P.Y.W., C.Y.K.C., V.W.H.Y., Y.C., and C.C.L.W. Drafting of the manuscript: C.Y. and C.C.L.W. Funding acquisition and project administration: C.Y. and C.C.L.W.

## Supporting information

Supporting Information

## Data Availability

The data that support the findings of this study are available from the corresponding author, upon reasonable request.
