# Supplementary material for: Genome‐Wide CRISPR/Cas9 Library Screening Revealed Dietary Restriction of Glutamine in Combination with Inhibition of Pyruvate Metabolism as Effective Liver Cancer Treatment
Source: Adv Sci (Weinh). 2022 Oct 30;9(34):2202104. doi: 10.1002/advs.202202104 (PMC9731711; doi:10.1002/advs.202202104)
Supplement: Supplementary file 1 — Supporting Information [file ADVS-9-2202104-s001.pdf]

ADVANCED SCIENCE
Open Access

Supporting Information

for *Adv. Sci.*, DOI 10.1002/advs.202202104

Genome-Wide CRISPR/Cas9 Library Screening Revealed Dietary Restriction of Glutamine in Combination with Inhibition of Pyruvate Metabolism as Effective Liver Cancer Treatment

*Chunxue Yang, Derek Lee, Misty Shuo Zhang, Aki Pui-Wah Tse, Lai Wei, Macus Hao-Ran Bao, Bowie Po-Yee Wong, Cerise Yuen-Ki Chan, Vincent Wai-Hin Yuen, Yiling Chen and Carmen Chak-Lui Wong**

Supplementary Materials

Genome-wide CRISPR/Cas9 library screening revealed dietary restriction of glutamine in combination with inhibition of pyruvate metabolism as effective liver cancer treatment

Chunxue Yang, Derek Lee, Misty Shuo Zhang, Aki Pui-Wah Tse, Lai Wei, Macus Hao-Ran Bao, Bowie Po-Yee Wong, Cerise Yuen-Ki Chan, Vincent Wai-Hin Yuen, Yiling Chen, Carmen Chak-Lui Wong[*]

Table of Contents:

Experimental Section—Cell apoptosis assay

Figure S1. Important pathways for adaptation to glutamine depletion.

Figure S2. Stable knockout of PDHA, PDHB, and PC in MHCC97L cells.

Figure S3. Mitochondrial potential of MHCC97L cells during the blockade of glutamine and pyruvate metabolism.

Figure S4. Cell viabilities and apoptosis of MHCC97L cells and MIHA cells treated with pharmaceutic treatments by PDH inhibitor or PC inhibitor.

Figure S5. Effects of PDHi, PCi, and glutamine deficient diet in mice with subcutaneous HCC derived from MHCC97L.

Figure S6. Effects of PDHi, PCi, and GLSi on subcutaneous HCC tumors.

Figure S7. Mouse body weights in orthotopic HCC model.

Table S1. Oligonucleotide sequences of the genes used in the study.

Experimental Section

Cell apoptosis assay

MHCC97L cells were seeded onto 6-well culture plates. For inhibitor treatments, MHCC97L cells were treated with PDH inhibitor (0, 10 or 100 μM) or PC inhibitor (0, 50, 500μM) under glutamine replete (4 mM) or depleted condition (0 mM) for 48h. All floating and adhered cells were collected and stained with propidium iodide (Calbiochem) and Annexin V (MBL International Corporation). Flow cytometric analysis was performed by BD LSRFortessa cell analyzer (BD Bioscience) and flow data were analyzed by Flowjo software (Flowjo).

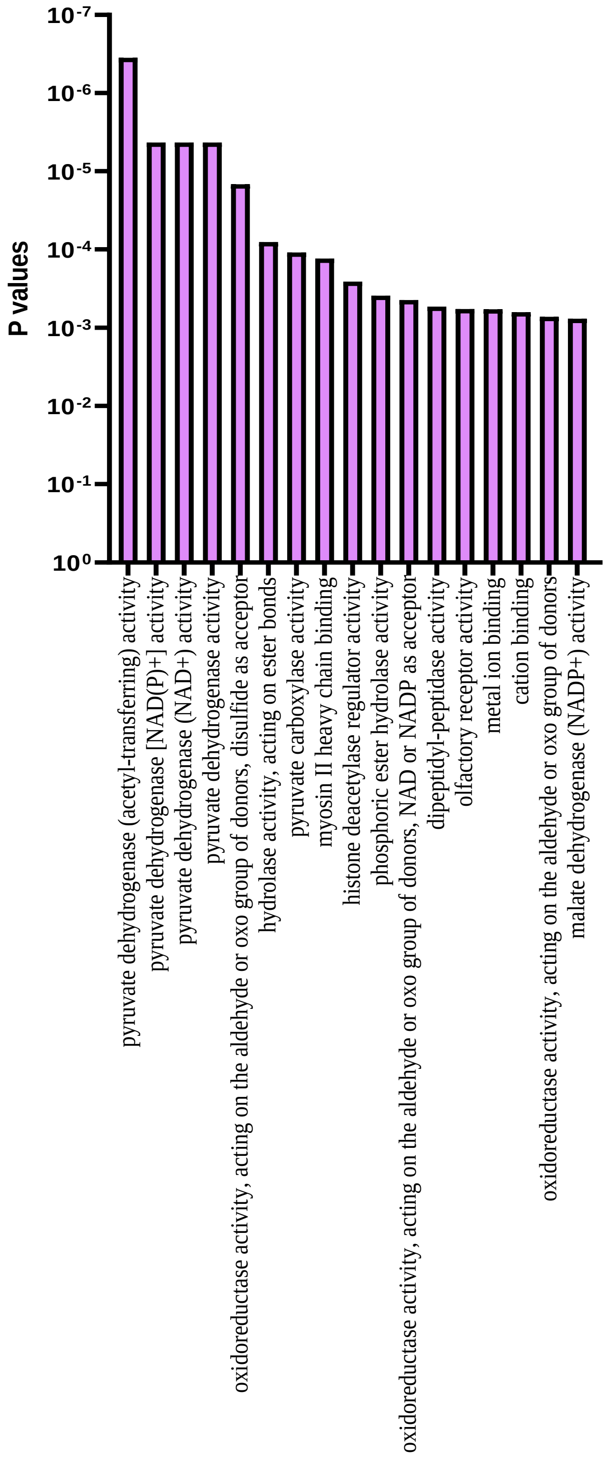

Figure S1. Important pathways for adaptation to glutamine depletion. GO enrichment analysis ranked pyruvate metabolism as the most important pathway for MHCC97L cell adaptation to glutamine depletion.

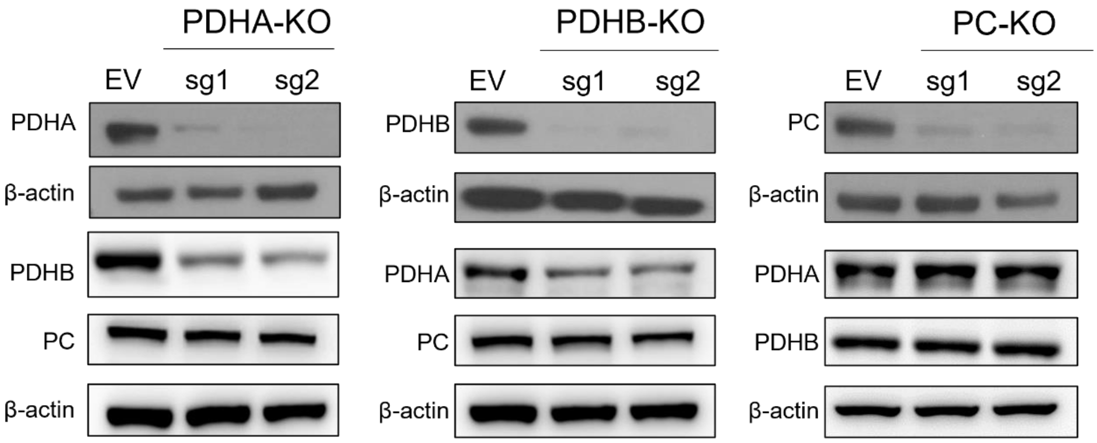

Figure S2. Stable knockout of PDHA, PDHB, and PC in MHCC97L cells. Knockout efficiency of PDHA, PDHB, and PC stable KO clones in MHCC97L was determined at the protein level by western blotting normalized to β-actin.

A

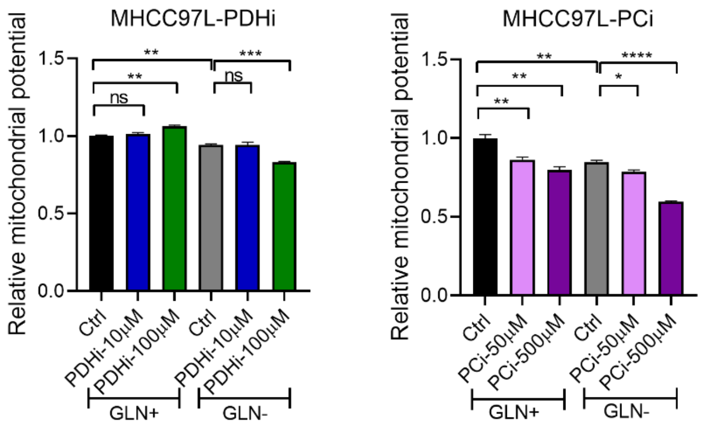

B

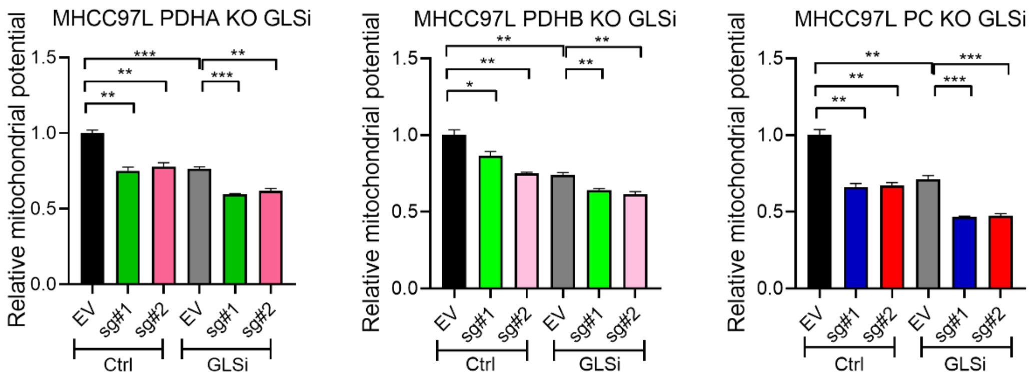

Figure S3. Mitochondrial potential of MHCC97L cells during the blockade of glutamine and pyruvate metabolism. (A) JC-1 staining demonstrated mitochondrial membrane potential of MHCC97L cells upon PDH or PC inhibitor treatment under glutamine depletion (GLN-, 0 mM) were significantly decreased compared to normal condition (GLN+, 4 mM). **(B)** JC-1 staining demonstrated mitochondrial membrane potential of PDHA, PDHB, and PC KO MHCC97L cells treated with GLS inhibitor (BPTES, 1 μM) were significantly decreased compared to control (Ctrl). *P < 0.05, **P < 0.01, ***P < 0.001, ****P < 0.0001 vs. Ctrl or EV as indicated. Student's t-test. Error bars indicate mean ± SEM (n = 3).

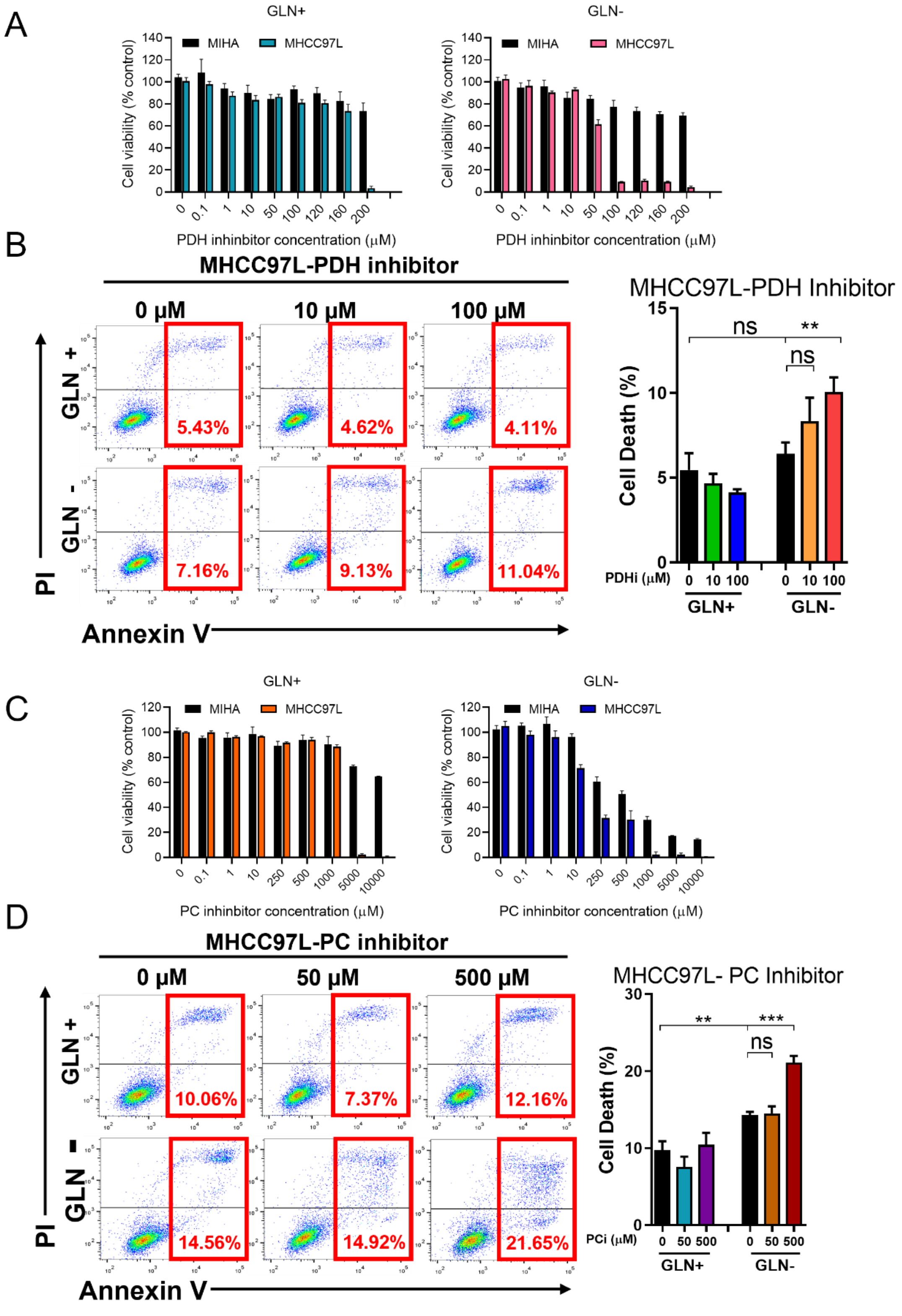

Figure S4. Cell viabilities and apoptosis of MHCC97L cells and MIHA cells treated with pharmaceutic treatments by PDH inhibitor or PC inhibitor. (A) Cell viabilities of MHCC97L cells and MIHA cells upon PDH inhibitor treatment under

glutamine replete (GLN+, 4 mM) and glutamine depletion (GLN-, 0 mM). **(B)** Apoptosis of MHCC97L cells treated with PDH inhibitor. **(C)** Cell viabilities of MHCC97L cells and MIHA cells upon PC inhibitor treatment under glutamine replete (GLN+, 4 mM) and glutamine depletion (GLN-, 0 mM). (D) Apoptosis of MHCC97L cells treated with PC inhibitor. Error bars indicate mean ± SEM (n = 3). *P<0.05, **P<0.01, ***P<0.001 vs control as indicated. Student's t-test. Error bars indicate mean ± SEM.

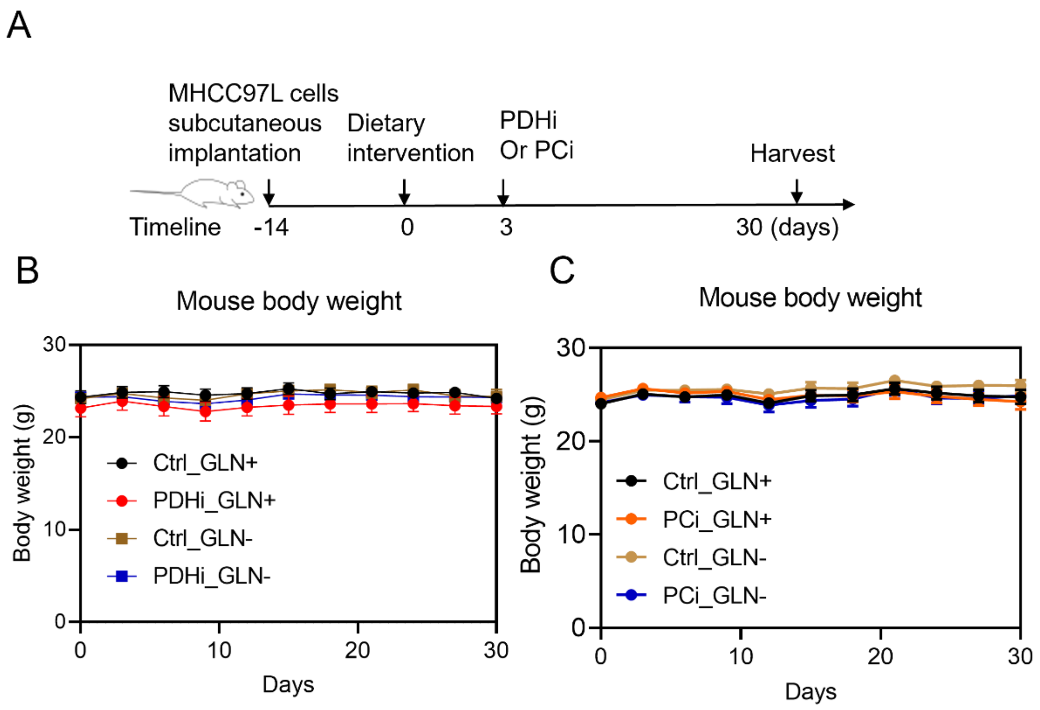

Figure S5. Effects of PDHi, PCi, and glutamine deficient diet in mice with subcutaneous HCC derived from MHCC97L. (A) Timeline of experiment. **(B-C)** Mouse body weights in groups receiving glutamine supplemented or depleted diets with vehicle control (Ctrl_GLN+/-) and (B) glutamine supplemented or depleted diets

with PDHi (PDHi_GLN+/-) (n=6) or (C) glutamine supplemented or deficient diets with (PCi_GLN+/-) (n=6). Error bars indicate mean ± SEM.

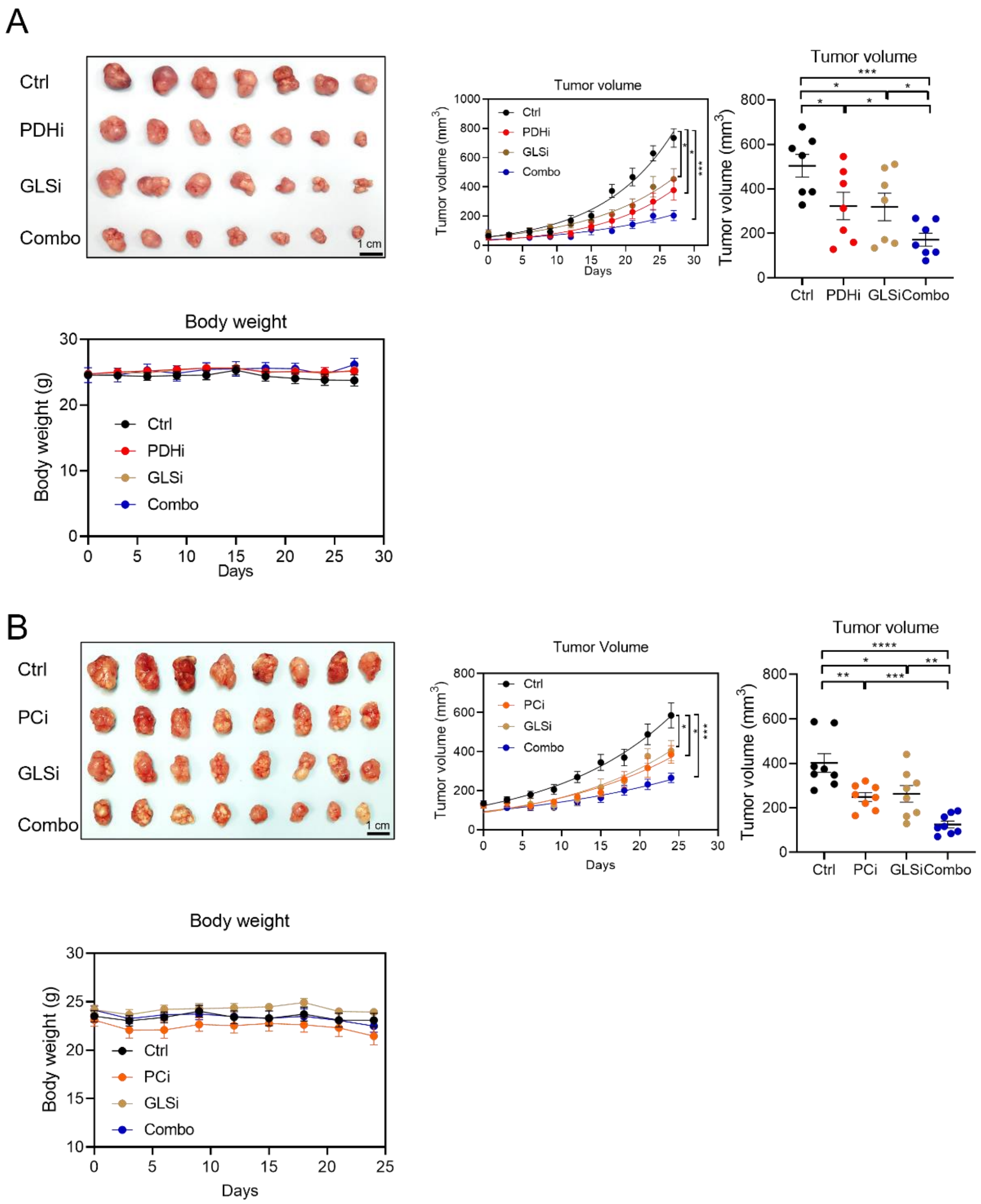

Figure S6. Effects of PDHi, PCi, and GLSi on subcutaneous HCC tumors. (A) Left: representative picture of MHCC97L subcutaneous tumors in mice administered with control (Ctrl), PDHi alone, GLSi alone, PDHi and GLSi combined treatment (Combo). Middle: tumor volume growth curve. Right: tumor volume of the harvested tumors. Bottom: Body weights of mice. (n=7). **(B)** Left: representative picture of MHCC97L subcutaneous tumors in mice administered with control (Ctrl), PCi alone,

GLSi alone, PCi and GLSi combined treatment (Combo). Middle: tumor volume growth curve. Right: tumor volume of the harvested tumors. Bottom: Body weights of mice. (n=8). *P < 0.05, **P < 0.01, ***P < 0.001, ****P < 0.0001 vs. Ctrl or as indicated. Student's t-test. Error bars indicate mean ± SEM.

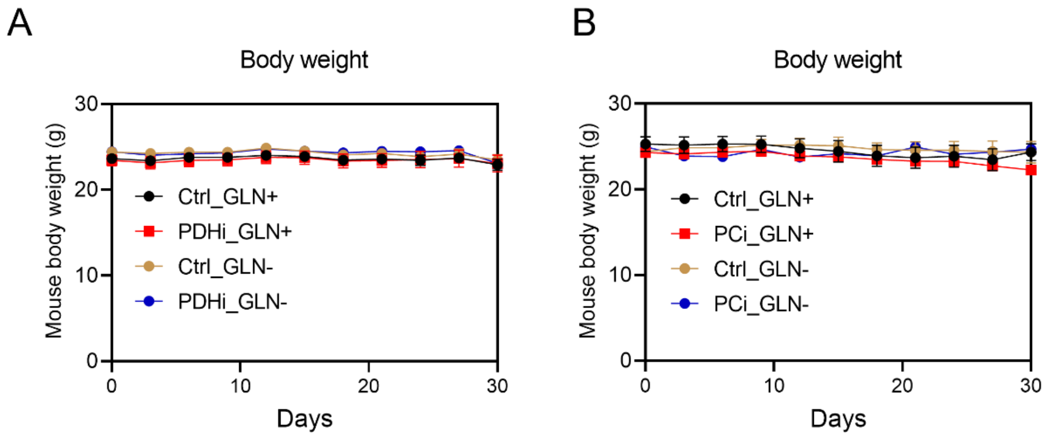

Figure S7. Mouse body weights in orthotopic HCC model. (A) Mouse body weights of groups receiving glutamine supplemented diet (Ctrl_GLN+), glutamine supplemented diet and PDHi (PDHi_GLN+), glutamine deficient diet (Ctrl_GLN-), glutamine deficient diet and PDHi (PDHi_GLN-). (B) Mouse body weights of groups received glutamine supplemented diet and PCi (PCi_GLN+), glutamine deficient diet and PCi (PCi_GLN-). (n=4). Error bars indicate mean ± SEM.

Name	Forward sequence 5’-3’	Reverse sequence 5’-3’
PDHA-sg#1	CACCGTGTGCGTCCGAGAGGCAACA	AAACTGTTGCCTCTCGGACGCACAC
PDHA-sg#2	CACCGTACCTTCCCAGATCTACAAT	AAACATTGTAGATCTGGGAAGGTAC
PDHB-sg#1	CACCGACCTGGATTGTTATCCCGAA	AAACTTCGGGATAACAATCCAGGTC
PDHB-sg#2	CACCGAAGGCCACCAGACATGTAGT	AAACACTACATGTCTGGTGGCCTTC
PC-sg#1	CACCGCTGAAGTTCCGAACAGTCCA	AAACTGGACTGTTCGGAACTTCAGC
PC-sg#2	CACCGACAGGTGTTCCCGTTGTCCC	AAACGGGACAACGGGAACACCTGTC

Table S1. Oligonucleotide sequences of the genes used in the study.
